# The Neurobiological Mechanisms of Generalized Anxiety Disorder and Chronic Stress

**DOI:** 10.1177/2470547017703993

**Published:** 2017-06-08

**Authors:** Michelle A. Patriquin, Sanjay J. Mathew

**Affiliations:** 1 Menninger Department of Psychiatry and Behavioral Sciences, Baylor College of Medicine, Houston, TX, USA; 2 The Menninger Clinic, Houston, TX, USA; 3 Michael E. Debakey VA Medical Center, Houston, TX, USA

**Keywords:** brain imaging/neuroimaging, genetics, biological markers, anxiety, stress

## Abstract

Two classification systems are now at the forefront of clinical psychiatric research: (1) Diagnostic and Statistical Manual, Fifth Edition and (2) the National Institutes of Mental Health Research Domain Criteria. Herein, we propose that these two classification systems are complementary rather than mutually exclusive, and when combined provide important information for understanding aspects of the pathophysiology related to Generalized Anxiety Disorder (GAD). The neurobiological literature for GAD and one relevant research domain criteria component, sustained threat, are reviewed from multiple units of analysis (genetic, neuroimaging, neuroendocrine, and psychophysiological). It is hypothesized that generating a comprehensive, biologically based understanding of the relationship between GAD, sustained threat, and the measureable units of analysis will provide information critical to design the most effective treatments.

## Introduction

Two—not necessarily opposing—classification systems are now at the forefront of clinical research: (1) Diagnostic and Statistical Manual, Fifth Edition (DSM-5^1^) and (2) the National Institutes of Mental Health (NIMH) Research Domain Criteria (RDoC^2–4^). RDoC provides a fresh perspective on new ways to approach anxiety research (e.g., through the construct of “potential threat”), but because of its clinical utility, the DSM-5 maintains its position as the diagnostic system that guides clinical practice (along with ICD-11). Yet, the biological and nosological characterization of DSM-5 anxiety disorders, particularly generalized anxiety disorder (GAD), and other comorbid disorders (e.g., major depressive disorder, MDD) are unclear and demonstrate high correlations and unreliability.^5–7^ It is hypothesized that an RDoC-based conceptualization and investigation may be able to provide answers to these shortcomings of the DSM-5 by developing a transdiagnostic, neurobiological model of anxiety that provides a more valid distinction from other psychiatric RDoC constructs (e.g., loss). Because of the primary clinical and research perspectives of the DSM-5 and RDoC classification systems, respectively, we provide a selective review of the neurobiological literature associated with GAD (DSM-5) and one relevant RDoC construct, chronic stress (“sustained threat”; RDoC). Notably, other RDoC constructs are also likely to be relevant to the neurobiology of GAD (e.g., potential threat, acute threat, arousal);^8^ however, sustained threat is used here to provide an illustration of how a connection between DSM-5 and RDoC classification systems can be made. The reader is referred to recent reviews of GAD neurobiology^9–12^ and RDoC^8^ for additional background information beyond the scope of this review.

### Generalized Anxiety Disorder

GAD is one of the most common psychiatric disorders, occurring in up to 21% of adults in their lifetime.^13^ As defined in the DSM-5, GAD is characterized by excessive anxiety and worry about a number of events or activities (e.g., work, school performance), which an individual finds difficult to control. The worry is impairing across varied contexts (e.g., work, home, and social). Symptoms which are required for diagnosis include feeling restless, being easily fatigued, difficulty concentrating or mind going blank, irritability, muscle tension, and sleep disturbance (note: only one symptom needs to be present in children). GAD has high rates of comorbidity, particularly between GAD and MDD that ranges from 40% to 98% in treatment studies.^14–18^ In fact, the GAD/MDD comorbidity may occur more often than MDD or GAD alone.^19^ In addition to high comorbidity, DSM-5 field trials have highlighted the poor diagnostic reliability of GAD (kappa = 0.34).^5^ Other findings indicate differences between illness predictors and symptoms of MDD and GAD.^20–23^ GAD is often a precursor of MDD and if GAD is treated effectively, it lowers the risk for development of MDD and some individuals with MDD never develop GAD.^24^ Because of these mixed findings, cross-cutting and distinct symptoms of GAD need to be investigated in accordance with transdiagnostic and RDoC perspectives; however, diagnostic categories (e.g., GAD vs. MDD) should also be examined due to their clinical relevance and adherence to the current nosological system (DSM-5).

Given the clinical ambiguities, it is not surprising that there is a limited biological information regarding how similar or different GAD is from other disorders (e.g., MDD). At the biological level, studies that have pursued a biological conceptualization from one unit of analysis (e.g., brain data) have often highlighted similar findings in depression and anxiety.^6,7,25^ For example, the amygdala has demonstrated increased activity in both anxiety and depression groups when compared to healthy controls,^6,25^ and twin studies indicate high genetic correlations between MDD and GAD (*r* = .74–1.0).^7,26,27^ However, MDD and GAD can be differentiated with respect to biology,^28–30^ particularly with resting-state functional magnetic resonance imaging (R-fMRI).^31^

As the current literature lacks clarity regarding the clinical and biological differences between GAD and other disorders, such as MDD, both DSM-5 (categorical approaches) and RDoC (cross-cutting constructs) approaches offer critical information for understanding the differences and overlap between GAD and other disorders. Thus, the present literature review examines aspects of the neurobiological literature from both a categorical (GAD) and dimensional (chronic stress) perspective in order to build an initial understanding of the diagnostic differences versus dimensional intersections (e.g., chronic stress across disorders) related to GAD.

### Chronic Stress

Chronic stress may be one cross-cutting construct or dimension (i.e., that occur across diagnostic categories defined by the DSM-5) related to GAD, as well as other diagnoses (such as highly related MDD). In general, a stress response may be adaptive in the short-term when faced with acute challenges.^32,33^ Over time, if an individual is subjected to short-term acute stressors that are repeatedly triggered, the resulting chronic maladaptive stress responses (i.e., chronic stress) may develop into interfering psychiatric difficulties or disorders.^34–36^ This resulting chronic stress impacts biological systems across multiple “units of analysis”—neuroendocrine, autonomic, and behavioral—when triggered by perceived or actual threat.^37^ Chronic stress can significantly impact the homeostatic biological system by increasing the allostatic load (i.e., effect of stress on the human body).^38,39^ Increased cumulative effects of stress on the human body (allostatic load) are linked to many adverse health consequences, including psychiatric illnesses such as GAD.

Individuals may be able to remain resilient (e.g., do not develop psychiatric disorders) to different amounts of cumulative stress based on their early-life experiences (e.g., trauma in early childhood^40^), hereditary factors, and stress history.^41–44^ For example, childhood trauma and life experiences increases the risk of onset and recurrence of depression and anxiety disorders.^45^ Childhood trauma, childhood life experiences, as well as genetic and environmental factors (e.g., stress in the family)^46,47^ may reduce an individual’s “tolerance” or resilience to increased allostatic load caused by repeated stressors; thus, increase the risk for the development of psychiatric disorders, including anxiety.

Early diagnosis (and treatment) of a psychiatric disorder also may contribute to an individual’s resilience and ability to cope with repeated stress. For example, carrying a diagnosis of depression or anxiety in childhood can increase risk of an individual being diagnosed with GAD in adulthood.^48^ Further, findings from a recent study found that more time in an episode of GAD (as well as MDD) was associated with the highest risk for experiencing suicidal thoughts and behaviors across adolescence and into adulthood.^49^ Not only was GAD diagnosis associated with the highest risk trajectory for suicidal thoughts and behaviors, broader anxiety symptoms were as well. Combined with the shocking 24% increase of suicide mortality in the past 15 years in the U.S.,^50^ these trajectory findings stress the impact of chronic stress and psychiatric diagnosis on future psychopathology.

The RDoC matrix^51^ breaks down cross-cutting constructs into five domains, including negative valence system domain, which includes constructs related to anxiety (e.g., potential threat and sustained threat). In the present review, we explore RDoC sustained threat as a highly relevant cross-cutting construct related to GAD and chronic stress. Although potential threat has been considered—and is highlighted in the RDoC matrix—as being related to anxiety, it is sustained threat (or the experience of chronic stress), whether potential or actual, that appears to have the most significant health consequences related to GAD (e.g., emergence of psychiatric disorders^34–36^). RDoC defines sustained threat as “an aversive emotional state caused by prolonged (i.e., weeks to months) exposure to internal and/or external condition(s), state(s), or stimuli that are adaptive to escape or avoid…” This description includes that the threat may be “actual or anticipated.” Alongside this description are the multilevel (molecules to behavior) neurobiological components hypothesized to be involved in the construct of sustained threat (see Table 1).

**Table 1.** RDoC sustained threat.

Molecules	Cells	Circuits	Physiology	Behavior
ACTH CRF HPA-axis hormones	Hippocampal Microglia Prefrontal	Attention network Dysregulation of amygdala reactivity Dysregulation of cingulate reactivity Habit systems Hypothalamic nuclei PVT Vigilance network	Dysregulated HPA Axis Error-related negativity	Anhedonia/decreased appetitive behavior Anxious arousal Attentional bias to threat Avoidance Decreased libido Helplessness behavior Increased conflict detection Increased perseverative behavior Memory retrieval deficits Punishment sensitivity

*Note.* ACTH, andrenocorticotropic hormone; CRF, corticotropin releasing factor; HPA, hypothalamic-pituitary-adrenal; PVT, paraventricular thalamic nucleus.

## Sustained Threat, GAD, Neurobiology: A Model

**Figure 1.**
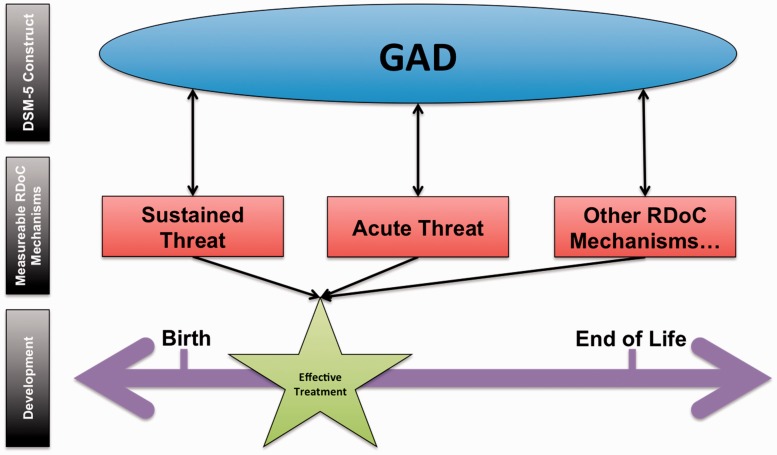
RDoC sustained threat and GAD conceptual model.

Here, we review the neurobiological literature associated with GAD and with the RDoC domain, sustained threat. As noted prior, this literature review is not intended to include an exhaustive review of all RDoC domains relevant to GAD (e.g., acute threat) but rather provide a demonstration of how both DSM-5 categorical perspectives and RDoC domains can be linked. Our proposed model (see Figure 1) includes three tiers: (1) DSM-5 construct (i.e., GAD), (2) RDoC mechanism, and (3) development. It is hypothesized that designing developmentally appropriate pharmacological and psychological treatments that target neurobiological mechanisms (e.g., bed nucleus of stria terminalis [BNST], elevated peripheral nervous system activity, *5-HTTLPR* short-allele) related to sustained threat, or other measurable RDoC mechanisms, may generalize and target the chronic anxiety demonstrated in individuals with GAD. For example, implementing a pharmacogenomics or metabolomics protocol to inform medication selection may improve pharmacotherapy treatment outcomes. Not only can neurobiological markers provide insight for developing personalized medicine protocols and offer biological objective markers of treatment efficacy, the implementation of successful treatment strategies may be dependent on timing. There are critical neurobiological developmental windows in childhood that may be most ideal to target to produce the best outcomes when treating neuropsychiatric disorders, including GAD (see “effective treatment” star placed on developmental timeline).^52^

Our conceptual model (Figure 1) organizes the relations between the relevant components discussed within the present review. GAD is intentionally placed within an oval to illustrate that the DSM-5 diagnoses are considered to be at the latent or at the construct level. Conversely, the RDoC mechanisms hypothesized to be related to GAD (as well as that GAD is related to these mechanisms; hence, bi-directional arrows between) are measurable (i.e., indicated by square shapes) through their specific units of analysis (i.e., genes, molecules, cells, circuits, physiology, behavior, self-reports, and paradigms). Our model highlights that it is the bi-directional relationship between DSM-5 constructs and RDoC mechanisms that will influence the development of effective treatments (not only one level or tier, such as an RDoC understanding only). Below, we review the DSM-5 (GAD) and one relevant RDoC dimension (sustained threat) to GAD in order to begin linking the levels in our model.

## GAD and Neurobiology

### Genetics and Epigenetics

It is estimated that genes contribute 30% to 50%^53,54^ to the development of an anxiety disorder. Conversely, development of an anxiety disorder due to non-genetic factors is approximately 50% to 70%. Environmental factors (e.g., stress, trauma, etc.) likely contribute to the development of anxiety disorders through epigenetic mechanisms that could impact the development of anxiety even beginning in utero. For example, mothers diagnosed with an anxiety disorder who did not receive medication for anxiety have demonstrated altered DNA methylation of the glucocorticoid receptor gene (NR3C1) promoter region in cord blood and the genome^55,56^ and may increase risk of their child developing an anxiety disorder.

Understanding the genetic and epigenetic mechanisms of GAD is important for building more comprehensive neurobiological conceptualizations to develop the most effective preventative and treatment strategies. Despite the potential impact of understanding these mechanisms, few human studies of anxiety disorders, including GAD, exist relative to animal studies.^12^ One genome-wide association study of GAD symptoms found genome-wide significant association between a single-nucleotide polymorphism (rs78602344) intronic to thrombospondin 2 (*THBS2*) and GAD symptoms in a population-based sample of Hispanic/Latino adults (*N* = 12,282; aged 18–74).^57^ Although not specific to GAD, a study of anxious versus non-anxious adults (*N* = 47; *M* age = 32.89) found that DNA methylation indicated that global methylation levels were higher in anxious adults relative to non-anxious adults^58^ (anxiety determined by Hospital Anxiety and Depression Scale-Anxiety scores). Hospital Anxiety and Depression Scale-Anxiety scores in the anxious group were also significantly correlated with the expression of the DNA methyltransferases DNMT1/3A, such that greater anxiety severity was associated with more DNA expression. Notably, the generalizability of the findings of this DNA methylation study is limited due to the small sample size.

### Neuroimaging

The disrupted coordination of brain activity has been proposed as one of the core neurobiological features of GAD,^59–61^ particularly reduced resting-state functional connectivity (RSFC) between the amygdala and prefrontal cortex (PFC) in both adults and adolescents with GAD.^62–64^ Yet, differences between GAD and healthy control groups, both in structural and functional neuroimaging, not only exists in frontolimbic areas but also in a downstream projection from the amygdala: the anterior cingulate cortex.^61^ Findings suggest that across adolescence and adulthood, decreased connectivity between the amygdala and PFC is associated with the diagnosis of GAD.^62–64^ The PFC is critical for the effective regulation of emotion, particularly ventromedial regions that appear to control negative emotion,^65^ such as anxiety. Fittingly, the ventrolateral PFC activity has been shown to increase (perhaps increasing regulation over limbic structures) following all pharmacological and psychological intervention in individuals with GAD.^66^

Frontolimbic structures not only impact anxiety-related responses in GAD but also have been demonstrated to differentially regulate autonomic mechanisms in individuals with GAD.^59^ Specifically, a recent neuroimaging study investigated the relationship between both central nervous system (PFC, amygdala) connectivity and autonomic (heart rate variability; HRV) in individuals with GAD pre- and post-perseverative cognition task.^59^ At baseline, individuals with GAD demonstrated less connectivity between the PFC and amygdala; however, after the perseverative cognition task, this PFC-amygdala connectivity increased in the GAD group whereas healthy controls decreased in this area (at a trend level). After the perseverative cognition task, HRV reduction (increase in autonomic activation) was predicted by decreased RSFC between the left amygdala-subgenual cingulate cortex and between the right amygdala-caudate nucleus. These findings provide initial evidence regarding specific neural modulation of differential psychophysiological responses between GAD and healthy control groups.

### Psychophysiology

In general, the psychophysiological literature demonstrates that individuals with GAD are in a more physiologically dysregulated state (e.g., low HRV, high heart rate, higher skin conductance levels) at baseline relative to controls^67,68^ and individuals with other anxiety disorders.^69^ Yet, this hyperarousal psychophysiological pattern in GAD is not universally observed.^70^ It is possible that psychophysiological differences between GAD and controls are context specific. For example, increased sympathetic nervous system activity and reduced HRV was observed in high trait worry individuals.^71,72^ Additionally, worrying prior to fearful imagery exposure appears to diminish any associated cardiovascular reactivity, rather than relaxation or neutral stimuli prior to fearful exposure that exposes heightened cardiovascular reactivity during a fearful exposure in GAD participants.^73,74^ Thus, it appears that relative to controls, GAD participants demonstrate heightened physiological arousal at baseline (e.g., higher heart rate), and these individuals are likely to demonstrate the greatest cardiovascular reactivity to an anxiety-provoking stimulus when asked to relax or view neutral stimuli. This may suggest that individuals with GAD demonstrate chronic physiological arousal at baseline and demonstrate an exaggerated physiological reactivity to fearful stimuli. Similarly, when compared to other anxiety disorders (i.e., social anxiety and GAD with panic attacks), GAD is characterized by elevated baseline startle, suggesting the presence of anxious thoughts in the absence of threat.^69^ Overall, it appears that relative to healthy controls and individuals with other anxiety disorders, individuals with GAD may exhibit a unique psychophysiological signature that is characterized by hypervigilant physiological response at baseline, as well as greater reactivity to threat.

### Summary: How findings fit into RDoC

Findings across genetics, neuroimaging, and psychophysiology indicate neurobiological signatures related to the diagnosis of GAD. When examined in more detail, findings appear more closely related to elevated baseline neurobiological states that are likely correlated with increased thoughts of worry/fear without the presence of an immediate threat. The durability of physiological and cognitive changes—whether related to actual or perceived threat—potentially contributes to the neurobiological differences highlighted above. This sustained threat (also the RDoC construct reviewed below) is one particularly relevant component in GAD. Please see Table 1 for the neurobiological and behavioral components hypothesized to be related to the RDoC construct of sustained threat.

## Sustained Threat and Neurobiology

### Genetics and Epigenetics

Prior findings suggest that serotonin (5-HT) regulation is related to emotional stability.^75^ Serotoninergic functioning and variability appear related to individual differences in responding to threat, particularly the variation of the serotonin transporter gene (*5-HTTLPR*), which is related to neural patterns associated with anxiety, including GAD. When shown fearful and angry faces, *5-HTTLPR* short-allele carriers show bilateral amygdala hyperactivation compared to *5-HTTLPR* long-allele homozygotes.^76–78^ Additionally, decreased connectivity between the amygdala and pregenual cingulate, as well as decreased gray matter volume in both brain structures, has been observed in *5-HTTLPR* short-allele carriers.^79^

Though studies have demonstrated the relationship between amygdala hyperactivity in *5-HTTLPR* short-allele carries (note: in healthy individuals with “normative” anxiety levels),^76,77^ some studies do not find the *5-HTTLPR* × stress interaction to be significant.^80,81^ *5-HTTLPR* short-allele carriers appear to be sensitive to environmental cues, which may contribute to anxious thinking when stressors/threats are present.^75^ This highlights a propensity of these individuals to be sensitive to acute threat cues in the environment, which may contribute to a hypervigilance or sustained threat over time. In support of this notion, *5-HTTLPR* short-allele carriers have been shown to have a positive correlation between life stress and resting activity of the amygdala and hippocampus,^82^ such that more lifetime stress is associated with more resting activity between the amygdala and hippocampus suggesting a sustained threat response.

### Neuroendocrinology and Neuroimaging/Neural Circuits

As highlighted in Table 1, there are important molecular-level components hypothesized to be related to sustained threat (e.g., corticotropin-releasing factor (CRF); adrenocorticotropic hormone; paraventricular nucleus; hypothalamic-pituitary-adrenal axis). These sustained threat molecular components are also key parts of the neuroendocrine system, which regulates the stress response. It is hypothesized that dysregulation of the neuroendocrine system contributes to anxiety. In particular, when in a state of threat (or anxiety), CRF is released from the paraventricular nucleus of the hypothalamus into the primary capillary plexus of the hypothalamo-hypophyseal portal system to stimulate the anterior pituitary to synthesize proopiomelancortin into the blood. Adrenocorticotropic hormone in the blood activates the synthesis and release of cortisol in humans from the adrenal glands of the kidneys. The hypothalamic-pituitary-adrenal axis is then “shut down” by this release of cortisol, or corticosterone, through the influence of the hypothalamus, pituitary, and hippocampus.

Changes in the neuroendocrine system related to sustained threat (i.e., chronic activation of the system described above) have been demonstrated in both animal and human studies. For example, in one preclinical study, rats received intraventricular infusions of CRF and were placed in a sustained threat (sustained exposure to bright light) or acute threat condition. Results demonstrated that longer duration (sustained threat) threat responses were related to the BNST and could be differentiated from acute threat response (via central nucleus of the amygdala) by their sensitivity to CRF-RI receptor antagonists.^83^ Similarly, in humans, the BNST has been associated with sustained threat/anxiety.^84^ An fMRI study examined neural response to negative and neutral pictures at predictable and unpredictable intervals. The amygdala demonstrated reactivity to negative pictures that was not dependent on predictability, whereas the BNST showed a linear increase of activation across conditions as a function of anxiety;^84^ thus, identifying the BNST as related to sustained threat/anxiety versus acute stress (amygdala).

### Psychophysiology

Psychophysiological responses related to sustained threat have been hypothesized to be measured by error-related negativity,^85^ which measures the fronto-centrally maximal negative deflection in the event-related potential, as individuals reporting high levels of worry have demonstrated enhanced error-related negativity during a stressor task (Stroop task).^86^ Further, peripheral nervous system literature highlights a heightened sensitivity to unpredictable threat in individuals with lower respiratory sinus arrhythmia (a measure of high frequency heart rate variability),^87^ which is consistent with the literature reviewed above that demonstrate that individuals with impairing levels of anxiety, as indicated by a diagnosis of GAD, demonstrate more sympathetic activity (e.g., higher heart rate, lower heart rate variability) at baseline and greater cardiovascular reactivity to unpredictable threat. Contrary to these cardiac findings, however, skin conductance responses to unpredictability appear to induce a physiological inhibition (e.g., possibly congruent with a “freezing” anxiety response) indicated by weaker skin conductance responses to cue (stimuli presented) and temporal (time intervals) unpredictability.^88^ In general, it appears that the unpredictability may maintain a sustained threat response that is correlated with a heightened baseline physiological state as well as elicit greater reactivity when a threat is presented.

## Conclusions

Across GAD (DSM-5) and sustained threat (RDoC-based) studies, findings provide initial evidence of the biological system overlap (see Table 2 for summary of findings). Results indicate that there is the potential for *5-HTTLPR* short allele carriers to be at-risk for development of GAD (likely over the course of repeated stress) and when anxiety/sustained threat responses reach a level of impairment, global methylation levels are increased (e.g., expression of DNMT1/3A). These genetic profiles or changes appear to influence RSFC, particularly involving amygdala. The chronicity of sustained threat—to the point of the development of GAD—appears to also affect baseline psychophysiological functioning (perhaps due to neural changes that result from genetic/epigenetic influences) and place an individual in a hypervigilant physiological state (high HR, lower HRV, higher SCR).

**Table 2.** GAD and RDoC sustained threat (ST) literature review findings.

Genetics/epigenetics		Neuroimaging/neural circuits		Psychophysiology	
GAD	ST	GAD	ST	GAD	ST
SNP rs78602344 related to GAD symptoms Global methylation higher in anxious vs. non-anxious (not diagnosed with GAD) Increased anxiety severity, increased expression of DNMT1/3A	*5-HTTLPR* short allele show bilateral amygdala hyperactivity to fearful and angry faces vs. *5-HTTLPR* long allele *5-HTTLPR* short allele sensitive to environmental cues *5-HTTLPR* short allele carriers show increased life stress with increased RSFC between amygdala and hippocampus	Decreased RFSC amygdala: PFC in adolescents and adults with GAD Decreased HRV predicts decreased RSFC between left amygdala: cingulate cortex and right amygdala: caudate nucleus during perseveration task	CRF infusion in rats during sustained threat activated BNST BNST linear increase in activation as function of anxiety (sustained threat)	Baseline GAD group: lower HRV, higher HR, higher SCR Increased cardiovascular reactivity during fearful exposure in GAD GAD has increased baseline startle response	Higher worry, greater event related negativity during stress Decreased HRV, increased sensitivity to unpredictable threat SCR weakened during threat (“freezing” response)

*Note.* BNST, bed nucleus of the stria terminalis; CRF, corticotropin releasing factor; GAD, Generalized Anxiety Disorder; HR, heart rate; HRV, heart rate variability; PFC, prefrontal cortex; RDoC, Research Domain Criteria; RSFC, resting-state functional connectivity; SCR, skin conductance response; SNP, single nucleotide polymorphism.

Despite the compatibility of results across biological systems, many studies that we reviewed investigated one biological system (e.g., genetics, genome-wide association study^57^); however, two studies did investigate the link between genetics/epigenetics and neuroimaging^82^ as well as neuroimaging and psychophysiology.^59^ The investigation of both GAD and sustained threat primarily from one biological system highlights a significant gap in the literature. In fact, novel computational methods have recently been designed to examine the link between genetics, neuroimaging, and clinical data^89^ and could be expanded to include psychophysiology (e.g., measuring psychophysiological response during R-fMRI protocols). In line with RDoC goals, study of the integration of the biological systems, and their integration with clinical data, will provide an important understanding of the biological processes that relate not only to psychiatric disorders but also to the cross-cutting constructs, such as sustained threat, that are hypothesized to provide critical evidence for the development of new, targeted treatments.^3^

## Future Directions

Going forward, it will be important to continue to seek a comprehensive, biologically based understanding of GAD, particularly in the context relevant RDoC anxiety constructs (e.g., sustained threat, acute threat). Further, treatments will need to be developed from a comprehensive (e.g., genes, brain, psychophysiology, behavior) neurobiological framework, and timing of treatment implementation will have to be empirically tested to understand the most impactful timing to produce the optimal treatment trajectory. This is particularly important for anxiety disorders, as they often emerge in school-age children when other key processes in neurodevelopment are occurring (e.g., synaptogenesis, myelination, and synaptic pruning).^52^ Other important considerations include the fluctuating nature of symptoms in clinical populations and inadequate power of many neuroimaging studies. These limitations stress the importance of study replication, which currently does not often occur across all of science.^90^ Data sharing (e.g., in line with priorities at NIMH), such as making study data sets publically available, can greatly aid investigators in their pursuit of study replication. Future directions in personalized medicine will not only need to take into account treatment targets and optimal timing (e.g., the developmental window from which to produce the quickest, most long-lasting, positive outcomes) but also carefully consider the limitations of this research that argue for replication and improved systems that encourage data sharing.
